# Real‐Time, Noninvasive Diagnosis of Bullous Pemphigoid: Diagnostic Performance of Line‐Field Confocal Optical Coherence Tomography

**DOI:** 10.1111/srt.70235

**Published:** 2025-09-24

**Authors:** Frank Friedrich Gellrich, Samal Rakhmetova, Claudia Günther, Stefan Beissert, Sarah Hobelsberger

**Affiliations:** ^1^ Department of Dermatology Faculty of Medicine and University Hospital Carl Gustav Carus Technische Universität Dresden Dresden Germany; ^2^ Skin Cancer Center at the University Cancer Center National Center for Tumor Diseases (NCT/UCC) Dresden Germany

**Keywords:** autoimmune blistering disease, bullous pemphigoid, eczema, line‐field confocal optical coherence tomography, non‐invasive diagnosis, real‐time imaging

## Abstract

**Background:**

Bullous pemphigoid (BP) is a common autoimmune blistering dermatosis often difficult to distinguish from other inflammatory conditions. Timely diagnosis is crucial for appropriate management. Line‐field confocal optical coherence tomography (LC‐OCT), a noninvasive imaging technique that uses an 800nm laser to generate high‐resolution vertical and horizontal cross‐sectional images of the skin, is evaluated as a diagnostic tool for BP.

**Materials and Methods:**

A prospective, single‐center study was conducted on 26 patients with suspected BP. LC‐OCT imaging, histopathology, and direct immunofluorescence (DIF) were performed. LC‐OCT images were assessed for the presence of key diagnostic features, including subepidermal cleavage and eczema criteria.

**Results:**

Based on histopathology, DIF, indirect immunofluorescence (IIF), and ELISA, the final diagnoses were 15 BP cases and 11 non‐BP cases. LC‐OCT correctly identified 12 of 15 BP cases and all 11 non‐BP cases, demonstrating a sensitivity of 0.8 and a specificity of 1.0. The presence of subepidermal cleavage was a key diagnostic criterion for BP (*p* = 0.000). The absence of alternating hypo‐ and hyper‐reflective layers (*p* = 0.000), thickened and disrupted stratum corneum (*p* = 0.014), spongiosis (*p* = 0.036), and thickened epidermis (*p* = 0.043), which occurred significantly less frequently in BP cases, further supported the diagnosis.

**Conclusion:**

LC‐OCT demonstrates high sensitivity and specificity in diagnosing BP, offering a rapid, point‐of‐care diagnostic approach. LC‐OCT can be used to evaluate unclear inflammatory skin conditions and guide further investigations. However, LC‐OCT has limitations in diagnosing non‐bullous stages of BP; thus, histology and DIF remain the gold standard for definitive diagnosis.

## 1 Introduction

Bullous pemphigoid (BP) is the most common autoimmune blistering dermatosis (AIBD), with an incidence ranging from 2.4 to 23 cases per million [1]. It predominantly affects elderly patients. After the age of 70, the incidence increases to 190 to 312 per million [2]. The cause is autoantibodies (IgG type) against the hemidesmosomal structural proteins BP180 and BP230 [3]. Before the appearance of blisters, eczematous or urticarial, intensely pruritic erythema usually develops. In this “non‐bullous stage” of BP, the disease may not be clinically distinguishable from eczema of other causes (e.g., atopic dermatitis, contact dermatitis, or urticaria) [4]. As the disease progresses, disseminated tense, serous, sometimes hemorrhagic blisters can appear on the skin [5] and mucous membranes [4, 6]. Studies show that BP has 1‐year mortality rates of up to 19% in the United States and the United Kingdom [7, 8]. In contrast to eczematous diseases, the treatment of BP often requires high‐potency topical steroids, such as clobetasol propionate, possibly in combination with systemic steroids [9]. Timely diagnosis of BP is crucial to alleviate patient suffering, reduce the need for systemic steroids, and lower mortality.

The diagnosis is made by combining clinical findings, histopathology, and direct immunofluorescence (DIF) [10]. Indirect immunofluorescence (IIF) [11] and an enzyme‐linked immunosorbent assay (ELISA) to identify autoantibodies [12] can provide additional diagnostic information. The choice of the correct biopsy location has a crucial impact on the results of histopathology and DIF. In lesional biopsies, the blister roof may be lost, while in perilesional biopsies, the split may not be captured. Hodge et al. found that only 54.3% of 81 biopsies with clinical suspicion of BP showed subepidermal splitting [13]. An eosinophilic infiltrate in the upper dermis is another important histological criterion of BP, especially in the non‐bullous stage [14]. For DIF, in contrast to histology, the lesional biopsy of non‐bullous skin provides the most reliable result and is 3.45 times more likely to be positive than in perilesional skin [10].

Disadvantages of these investigations include the necessary intervention on the patient (biopsy and blood sampling), the associated costs, and the turnaround time for test results, which typically ranges from 1 day to 1 week. Nowadays, various modern imaging methods are available that allow in vivo diagnostics of the skin in real time for both neoplastic [15] and inflammatory diseases [16]. One of these techniques is LC‐OCT [17]. LC‐OCT combines the principles of conventional optical coherence tomography (OCT) and reflectance confocal microscopy (RCM) [18, 19]. Using an 800 nm laser, LC‐OCT generates vertical and horizontal cross‐sectional images of the skin up to a penetration depth of 400 µm with a lateral resolution of 1.3 µm, allowing the visualization of individual cells [17]. Tognetti et al. defined various patterns that can be used to diagnose and differentiate various AIBDs, including BP, pemphigus group, mucous membrane pemphigoid, dermatitis herpetiformis, linear IgA dermatosis, and epidermolysis bullosa acquisita [17]. Inflammatory dermatoses, such as atopic dermatitis, psoriasis, contact dermatitis, lichen planus, and infectious diseases, can also be diagnosed and differentiated using LC‐OCT [20, 21, 22]. The aim of this study is to evaluate the effectiveness of using LC‐OCT as a bedside test in the diagnosis of BP and to facilitate the identification of the optimal location for a biopsy.

## 2 Material and Methods

### 2.1 Study Population

Patients presenting with suspected BP at the Department of Dermatology, University Hospital Carl Gustav Carus, between February 24, 2023, and April 12, 2024, were enrolled in this single‐center, prospective study. A detailed medical history was obtained, and photographic documentation was performed. Subsequently, a representative skin area was identified for LC‐OCT imaging and two punch biopsies for DIF and histopathological examination. Blood samples were collected as part of routine diagnostics. The study protocol was explained to the patients by the examining physician, and written informed consent was obtained from all participants. The study protocol adhered to the principles of the Declaration of Helsinki and was approved by the Ethics Committee of the TU Dresden (BO‐EK‐557122022).

### 2.2 LC‐OCT Examination

LC‐OCT examination was performed using a commercially available handheld LC‐OCT probe (deepLive, DAMAE Medical, Paris, France). The device utilizes a Class 1 Supercontinuum laser with a wavelength range of 600–900 nm, providing an axial and lateral resolution of < 1.3 µm and a penetration depth of > 400 µm. It is capable of acquiring a 3D stack with dimensions of 1.2 × 0.5 × 0.5 mm (length, width, thickness). Initially, a representative skin area with a small, intact blister was identified to capture the entire blister, if possible. If complete visualization of the blister was not feasible, the blister edge was imaged to capture the transition from intact epidermis to the developing cleavage. In the absence of a blister, a hyperemic papule was selected. The area of interest was marked with a skin marker (Edding 780 creative 0.8 mm). A drop of paraffin oil was applied to the tip of the probe to minimize difference of optical index between the probe and the epidermis. Subsequently, at least one 3D stack was acquired for each patient. Image acquisition was performed by a PhD student (S.R.) under the direct supervision of a board‐certified dermatologist (S.H.). Additional images or video recordings were obtained based on the joint decision of the PhD student and the examining physician. Image analysis was performed by the PhD student and subsequently reviewed by an experienced physician using the software integrated into the device. The images of each patient were evaluated for the presence of a blister, the intra‐ or sub‐epidermal location of the blister, and the filling of the blister lumen with fibrin, erythrocytes, or inflammatory cells [17]. A quantitative grading of blister contents (e.g., “cell‐poor” vs. “cell‐rich”) was deliberately not performed to avoid subjective interpretation and because the limited number of blistering lesions in the cohort was insufficient for a statistically meaningful sub‐group analysis. The epidermis was assessed for inflammatory cells, the degree of spongiosis, disrupted honeycomb pattern, intraepidermal vesicles, thickened and disrupted stratum corneum, alternating hypo‐ and hyperreflective layers, and thickened epidermis [22]. In the dermis, the patterns of loosening, round vessels, linear vessels, and inflammatory cells were examined. Finally, based on these features, an overall assessment was made to determine whether BP was present or not. Since BP is the most common AIBD and this study aims to determine the sensitivity and specificity of the examination, the study groups were divided into BP and non‐BP. Two‐dimensional images for this publication were extracted from the 3D stack using 3DSlicer software, version 4.11.0‐2020‐07‐08 r29209.

### 2.3 Laboratory Tests

For histological examination and DIF, 4‐mm punch biopsies were performed on lesional and perilesional skin under local anesthesia with 2% lidocaine. The suitability of the lesional area was confirmed prior to biopsy using LC‐OCT imaging. Additionally, all patients underwent blood sampling for IIF and ELISA. Salt‐split skin IIF was not routinely performed as it is reserved for cases with negative BP180/BP230 serology but ongoing suspicion of a subepidermal autoimmune blistering disease, which was not the case for the patients in this cohort.

### 2.4 Statistical Analysis

Statistical analysis was performed using IBM SPSS Statistics, version 26. Fisher's exact test was used to assess the statistical significance of differences in documented parameters between the groups when comparing two categorical variables. The Chi‐squared test was used for comparisons involving more than two groups. The significance level was set at 0.05.

## 3 Results

### 3.1 Study Population

Twenty‐seven patients with suspected BP were initially enrolled in this study. One patient was excluded because DIF results were uninterpretable due to technical issues; therefore, 26 patients were included in the analysis. Based on the combined results of histopathology, DIF, IIF, and ELISA, the final diagnoses were 15 BP cases and 11 non‐BP cases, including nine pruritic eczemas, one drug eruption, and one dermatitis herpetiformis. DIF findings were consistent with the final diagnosis in all cases. In all diagnosed BP cases, ELISA detected antibodies against BP180; in 8 of 15 cases, antibodies against BP230 were also detected. In the case diagnosed as dermatitis herpetiformis, histopathology showed no cleavage and could not differentiate between eczema and dermatitis herpetiformis. IIF showed no endomysial antibodies. The diagnosis was established based on the focal presence of IgA antibodies along the basement membrane zone and a granular IgA‐pattern in the dermal papillae in the DIF. The patients ranged in age from 70 to 96 years (average 82.2 ± 6.4 SD), with 13 females and 13 males.

### 3.2 LC‐OCT

Our results are categorized based on their location within the skin: cleavage, epidermis, and dermis.

#### 3.2.1 Cleavage

**FIGURE 1:**
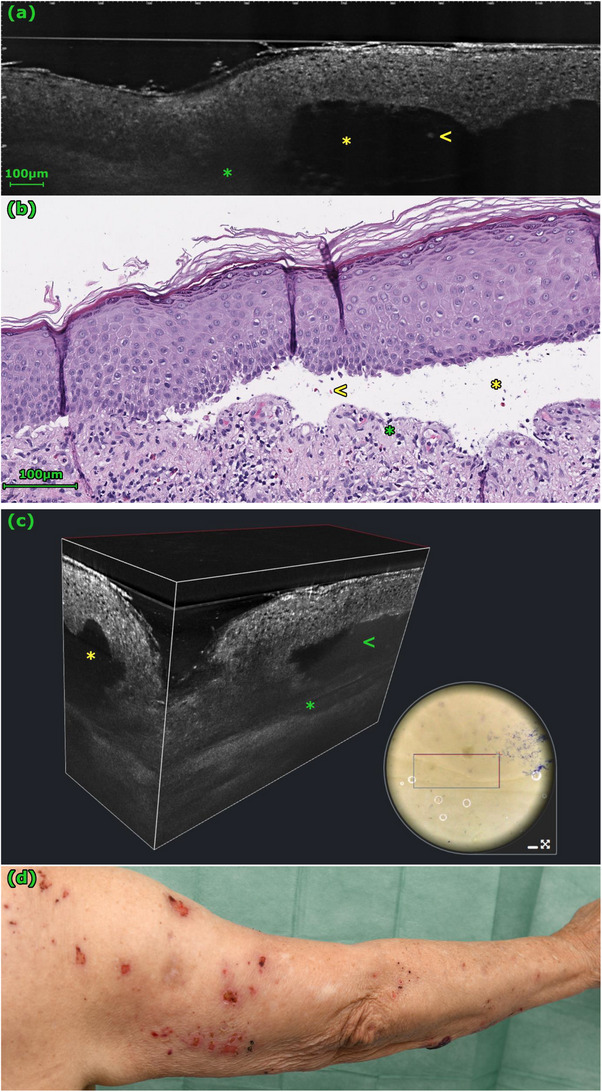
The subepidermal cleavage (yellow *) in this case of bullous pemphigoid is clearly visible in the LC‐OCT image (a) and the histology (b). In the 3D stack, it is evident that the epidermis above the cleavage (yellow *) is intact. The blisters are filled with erythrocytes and inflammatory cells (yellow <) and homogeneous fibrin deposits (green <). The loosened, homogeneous hyporeflective upper dermis in the LC‐OCT image (a and c, green *) corresponds to dermal edema (b, green *). In the absence of characteristic tense blisters, bullous pemphigoid can be clinically difficult to differentiate clinically from eczema. In addition to the numerous erosions, this picture shows a hemorrhagic blister on the elbow (d).

**FIGURE 2:**
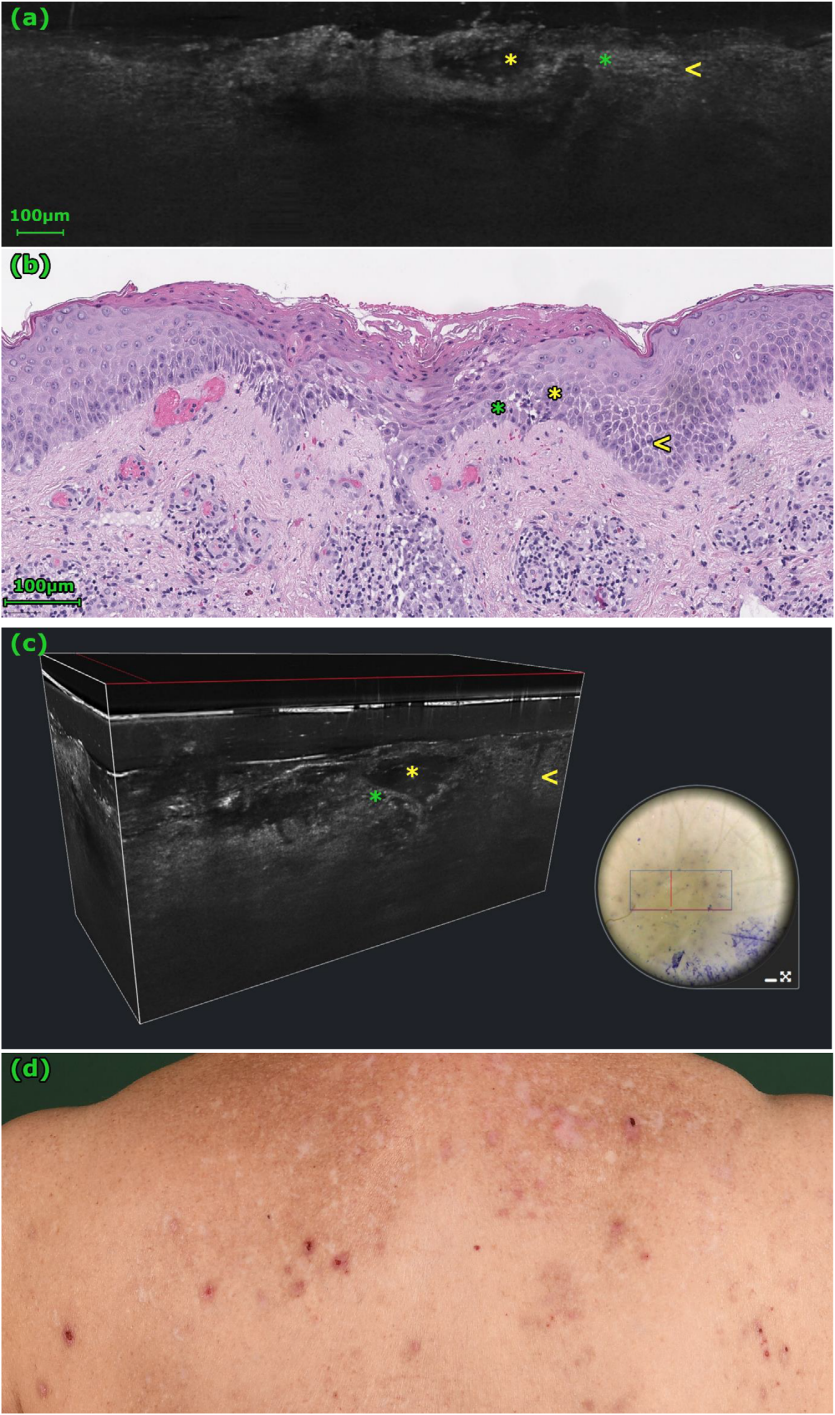
These images depict eczema. In the 2D LC‐OCT image (a) and the 3D stack (c), the intraepidermal location of the vesicle with underlying keratinocytes is clearly visible (yellow *). In the histology (b), the vesicle is not fully captured. However, pronounced spongiosis (yellow <) with wide white intercellular lines transitioning into incipient spongiotic vesicle formation (yellow *) is noticeable. The spongiosis is visible in the LC‐OCT images (a, c) as diverging keratinocyte cell nuclei (hyporeflective points) with widened intercellular space appearing (whitish gray thick network) (yellow <). Intraepidermally aggregated inflammatory cells are visible in the histology (b) (green *). These are also visible as hyperreflective points in the LC‐OCT images (a, c) (green *). The clinical picture (d) shows erosive papules that are difficult to distinguish from BP.

In our study, blisters were significantly more frequently observed in LC‐OCT images of BP cases than in eczema cases (*p* = 0.001) (Table 1). In 12 of 15 confirmed BP cases, a split formation was visualized at the dermoepidermal junction (DEJ) (Table 1, Figure 1). In only one of 11 eczema cases, a blister was visible; it was located in the epidermis (Table 1, Figure 2). Fibrin, erythrocytes, and inflammatory cells were found in almost all blisters and were not useful for differential diagnosis between blistering diseases in the present study (Table 1).

**TABLE 1:** Comparison of Line‐Field Confocal Optical Coherence Tomography (LC‐OCT) features between patients with bullous pemphigoid (BP, *n* = 15) and non‐BP conditions (*n* = 11).

	Bullous pemphigoid	Not bullous pemphigoid	*p* value
Number (*n*)	15	11	
**Assessment of blister**			
Blister existing	12/15 (80%)	1/11 (9.1%)	**0.001**
Blister at DEJ	12/15 (80%)	0/11 (0%)	**0.000**
Blister intraepidermal	0/15 (0%)	1/11 (9%)	0.423
Blister fill: fibrin deposition (homogeneous material mildly reflective)	12/12 (100%)	1/1 (100%)	—
Filling fill: erythrocytes (moderately reflective, roundish, floating elements)	11/12 (92%)	0/1 (0%)	0.154
Blister slightly gray, filled with inflammatory cells	12/12 (100%)	1/1 (100%)	—
**Assessment of epidermis**			
Inflammatory cells	15/15 (100%)	8/11 (73%)	0.630
Spongiosis None/low moderate/strong	8/15 (53%) 7/15 (47%)	1/11 (9%) 10/11 (91%)	**0.036**
Disrupted honeycomb‐pattern	12/15 (80%)	11/11 (100%)	0.238
Intraepidermal vesicles (dark, roundish areas)	0/15 (0%)	3/11 (27%)	0.063
Thickened and disrupted stratum corneum	6/15 (40%)	10/11 (91%)	**0.014**
Alternating hypo‐ and hyperreflective layers	0/15 (0%)	8/11 (73%)	**0.000**
Thickened epidermis (acanthosis)	3/15 (20%)	7/11 (64%)	**0.043**
**Assessment of dermis**			
Loosening	9/12 (75%) not assessable (3)	2/11 (18%)	**0.012**
Round vessels	7/10 (70%) not assessable (5)	6/10 (60%) not assessable (1)	1.000
Linear vessels	5/10 (50%) not assessable (5)	3/10 (30%) not assessable (1)	0.650
Inflammatory cells	8/9 (89%) not assessable (6)	6/10 (60%) not assessable (1)	0.303

*Note*: Data are presented as the number of cases exhibiting the feature/total number of assessed cases (percentage). *p*‐values were calculated using Fisher's exact test to determine the statistical significance of differences between the groups (significance level *p* < 0.05). Features are categorized by location: blister characteristics, epidermal findings, and dermal findings. For some dermal features, assessment was not possible in all cases due to imaging limitations; these instances are noted in the table.

Abbreviation: DEJ, Dermoepidermal junction.

#### 3.2.2 Epidermis

**FIGURE 3:**
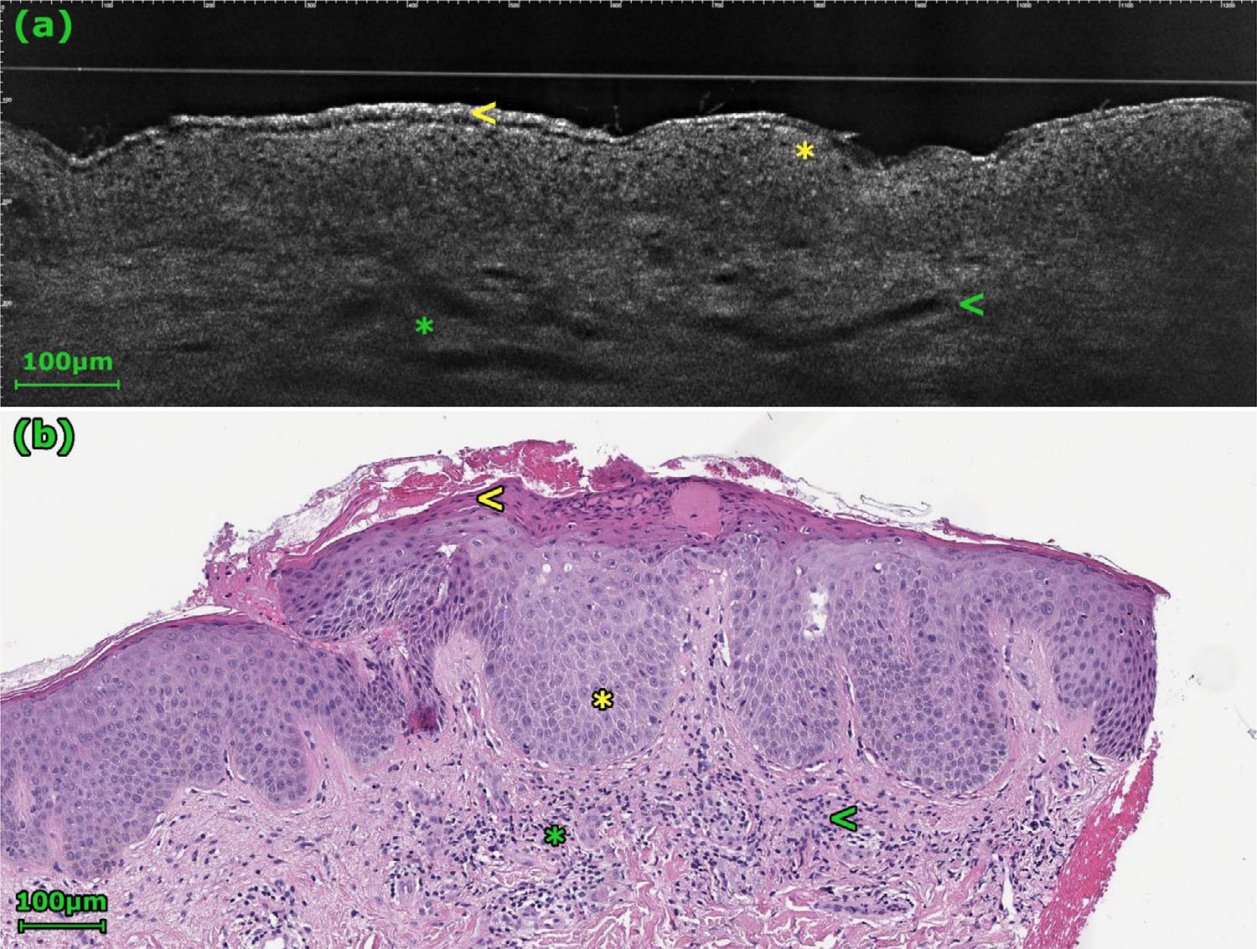
In this case of eczema, spongiosis is again visible (yellow *). The variably sized hyporeflective points correspond to a disrupted honeycomb pattern (a, yellow *). The stratum corneum is thickened and disrupted, showing alternating hypo‐ and hyperreflective layers in the LC‐OCT image (a) (yellow <). Round, tortuous, and partially dilated vessels are found in the dermis (green *). The dermal inflammatory cells (green <) are more difficult to visualize in the LC‐OCT images (a) due to their deeper location compared to the histology (b).

In the present study, moderate or strong spongiosis was significantly more frequently observed in eczema cases (91% vs. 47%, *p* = 0.036) (Table 1, Figures 2 and 3). A thickened and disrupted stratum corneum (91% vs. 40%, *p* = 0.014), alternating hypo‐ and hyperreflective layers (73% vs. 0%, *p* = 0.000), and a thickened epidermis (acanthosis) (64% vs. 20%, *p* = 0.043) were also significantly more frequent in eczema cases (Table 1, Figures 2 and 3). The disrupted honeycomb pattern in the epidermis was frequently observed in both eczema and BP cases, while intraepidermal vesicles were rare in both conditions (Table 1). Therefore, neither of these patterns proved suitable for differentiating between eczema and BP cases in the present study.

#### 3.2.3 Dermis

Due to the limited penetration depth of LC‐OCT, the various dermal patterns could not be assessed in all cases in our study. However, a loosening of the dermis was observed significantly more frequently in cases of BP (75% vs. 18%, *p* = 0.012) (Table 1, Figure 2). In contrast, the distribution of roundish and linear vessels, as well as inflammatory cells, did not show a significant difference between the two groups (Table 1, Figure 3).

#### 3.2.4 LC‐OCT‐Accuracy

For the diagnosis of BP, the presence of a subepidermal cleavage and the absence of eczema criteria—namely spongiosis, thickened and disrupted stratum corneum, alternating hypo‐ and hyperreflective layers, and thickened epidermis, and (to a lesser extent) loosening of the dermis—showed significant differences compared to non‐BP conditions (Table 1). Based on the observed patterns, the examining physician made an overall assessment using LC‐OCT images to determine whether BP was present or not. Using LC‐OCT, 12 out of 15 ultimately diagnosed BPs and 11 out of 11 non‐BPs were correctly assessed. The sensitivity of the LC‐OCT examination was 0.8, the specificity was 1.0, and the diagnostic accuracy was 0.88, with a positive predictive value of 1.00 and a negative predictive value of 0.76.

## 4 Discussion

BP, being the most common AIBD, can often be clinically difficult to distinguish from inflammatory dermatoses, particularly chronic eczema. However, timely diagnosis is crucial for initiating appropriate therapy early on. In the present study, LC‐OCT examination proved well‐suited to support diagnostics in cases of suspected BP.

To interpret our findings, we correlated them with established LC‐OCT criteria, which have been previously described in the literature [17, 22]. Split formation in the skin is always pathological. BP leads to subepidermal split formation, often with an intact overlying epidermis, while severe eczema can cause intraepidermal spongiotic vesicles [17]. In LC‐OCT, these different locations can be distinguished. In our study, the presence of a cleavage at the dermoepidermal junction was a key diagnostic criterion for BP. This confirms previous observations that the location of the cleavage can be well assessed with LC‐OCT [17]. Blisters in BP are typically filled with fibrin, erythrocytes, and inflammatory cells, appearing in LC‐OCT as a mix of homogeneous, mildly reflective material and moderately reflective, roundish floating elements [17]. We observed these features consistently, but they were not useful for differentiating from the single case of intraepidermal blistering in our cohort.

Epidermal changes are prominent in chronic inflammatory diseases. In eczema, inflammation leads to spongiosis (widened intercellular spaces), which appears in LC‐OCT as a thickened, white honeycomb network [22]. This can progress to intraepidermal microvesicles, which appear in LC‐OCT as dark roundish areas, significantly smaller than a blister. Intraepidermal cleavage in pemphigus vulgaris should be considered in the differential diagnosis. Other features of eczema include a thickened and disrupted stratum corneum and alternating hypo‐ and hyperreflective layers, corresponding to serum crusts [22]. In BP, the epidermis often appears less affected [23]. Our findings align with this, as alternating hypo‐ and hyper‐reflective layers were exclusive to non‐BP cases, and features like significant spongiosis, disrupted stratum corneum, and epidermal thickening were significantly more frequent in non‐BP conditions.

Dermal changes can also be observed with LC‐OCT, although visualization is often limited by penetration depth. In inflammatory conditions, dermal edema manifests as a loosening of the dermis (diffuse darkening), and vessels may appear dilated and tortuous. In our study, a loosening of the dermis was indeed observed significantly more frequently in cases of BP, likely corresponding to the prominent edema that accompanies blister formation. Other dermal features like vascular patterns or inflammatory infiltrates could not be reliably differentiated between the groups, underscoring the limitations of dermal assessment with this technology.

Notably, the high specificity of 1.0 meant that the diagnosis of BP was ruled out in all non‐BP conditions. “Non‐bullous stages” of BP can be challenging to diagnose with LC‐OCT due to the absence of blisters. In our cohort, the three BP cases without visible blisters were not correctly identified. In such cases, the absence of eczema criteria may be indicative, but distinguishing non‐bullous BP from eczema remains a significant challenge for this imaging modality.

Advantages of LC‐OCT examination include its ease of performance and real‐time evaluation. Correctly capturing the blister in histology can be challenging, as the blister roof may be lost during histological processing, may not be captured on representative cross‐sections, or may not be encountered during biopsy collection. However, the assessment of biopsy quality is only possible after at least 1 day, and sometimes up to a week. In contrast, LC‐OCT examinations can be repeated as often as necessary. In addition to histology, DIF is also a gold standard in the diagnosis of BP. For both investigations, the choice of the correct location of the biopsy is crucial. While tissue for histology should be taken perilesionally to capture the transition from intact skin to blister formation, DIF should be performed lesionally on non‐bullous skin for the best results. With the aid of the LC‐OCT examination, it was easier to determine the optimal biopsy location.

Previous studies describe the use of RCM in the diagnosis of bullous skin diseases [24, 25, 26]. RCM generates horizontal cross‐sectional images of the skin, while LC‐OCT additionally produces vertical cross‐sectional images. Horizontal cleavages can be better assessed in vertical cross‐sectional images because the imaging plane traverses the cleavage plane perpendicularly. Furthermore, the 3D stack of LC‐OCT simplifies the assessment of the blister location, as the diagnostically important transition from attached skin to cleavage can be found very easily. However, a direct head‐to‐head study comparing the diagnostic accuracy of LC‐OCT and RCM for AIBDs has not yet been performed and would be a valuable future endeavor. In addition to RCM, the use of OCT for the diagnosis of AIBDs has also been investigated [27, 28]. In contrast to RCM and LC‐OCT, OCT does not achieve cellular resolution but has a deeper penetration depth. While the blister can be visualized, cellular criteria such as spongiosis or inflammatory cells cannot be depicted. Accordingly, the benefit of OCT in this context appears lower and is recommended in combination with RCM [27]. In contrast, LC‐OCT combines the advantages of both imaging techniques. The growing potential of LC‐OCT in the diagnostic work‐up of bullous diseases has also been acknowledged in recent reviews [16].

The application of LC‐OCT requires experience and training. The device's probe is relatively heavy and must be held steady with one hand while simultaneously operating several buttons, while the other hand stabilizes the skin and probe head. It takes time to familiarize oneself with the evaluation of the black and white cross‐sectional images. However, prior knowledge in histology, RCM, or OCT can be readily transferred to LC‐OCT interpretation.

A limitation of this study is the small sample size, particularly the limited number (*n* = 3) of BPs in the “non‐bullous stage.” These BPs were poorly recognized with LC‐OCT and, depending on their proportion in the study population, can significantly affect the sensitivity of the examination. Furthermore, our study focused on the common clinical differential of BP versus eczema. While previous work has described qualitative LC‐OCT criteria for other subepidermal AIBDs [17], our cohort did not include entities such as epidermolysis bullosa acquisita or linear IgA dermatosis. Therefore, future prospective studies are needed to statistically validate these criteria and quantify the diagnostic performance (e.g., sensitivity, specificity) of LC‐OCT in differentiating between this broader spectrum of blistering diseases. The limited assessability of the dermis is also a limitation of the LC‐OCT examination. In histology, eosinophilic granulocytes are an important indicator of BP in the “non‐bullous stage” [23]. These cells cannot be identified using LC‐OCT.

## 5 Conclusion

The present study demonstrates that LC‐OCT has high sensitivity and specificity in the diagnosis of BP. The ease and speed of application, combined with the generation of vertical cross‐sectional images, make LC‐OCT a valuable tool for rapid, point‐of‐care diagnosis. A potential diagnostic algorithm could involve using LC‐OCT to evaluate unclear inflammatory skin conditions and, if cleavage is detected, initiating further investigations specifically for BP. However, it is important to acknowledge that LC‐OCT has limitations in diagnosing non‐bullous stages of BP. Therefore, histology and DIF remain the gold standard for definitive diagnosis.

## Conflicts of Interest

The authors declare no potential conflicts of interest with respect to the research, authorship, and/or publication of this article.

## Data Availability

The data collected as part of this study are available on request from the corresponding author.
